# Evaluation of the safety of using harmonic scalpel during laparoscopic cholecystectomy in children: A preliminary report

**DOI:** 10.3389/fped.2022.998106

**Published:** 2022-08-29

**Authors:** Ahmed Aboelela, Mohamed Abouheba, Ahmed Khairi, Mostafa Kotb

**Affiliations:** Pediatric Surgery Unit, Faculty of Medicine Alexandria University, Alexandria, Egypt

**Keywords:** children, cholecystectomy, cystic duct, harmonic scalpel, laparoscopic surgery

## Abstract

**Background and objective:**

In spite of being one of the most common surgical procedures performed in adults, laparoscopic cholecystectomy (LC) is relatively uncommon in the pediatric age group. Most surgeons prefer to dissect the cystic duct using a monopolar electrosurgical hook and occlude it with simple metal clips. Although the safety of using the ultrasonically-activated shears, e.g., harmonic scalpel for dissection of the gallbladder is confirmed in many studies, its efficacy in the closure of the cystic artery and duct in adults is still debatable. Furthermore, very few reports studied its safety in children during LC. The aim of our work is to study the safety and efficacy of ultrasonic shears in controlling the cystic duct and artery during LC in children.

**Materials and methods:**

A prospective study was conducted from May 2017 to April 2020, where all children having symptomatic gallbladder stone disease were included in the study. HS was used as a sole instrument in gallbladder dissection as well as in controlling cystic duct and artery. No metal clips or sutures were used throughout the procedure.

**Results:**

A total of forty-two children having symptomatic gallstone disease were included in the study. The main indication for LC was hemolytic anemia. Their age ranged from 3 to 13 years with a mean of 8.4 ± 3.25 years. All operations were completed laparoscopically, i.e., no conversion to open surgery was needed. The mean operative time was 40 ± 10.42 min. There were no intraoperative complications apart from gall bladder perforation in two cases during dissection from the liver bed while the postoperative recovery was smooth in all patients. Patients started oral feeding after 11.30 ± 3.01 h. The mean time for discharge was 25.47 ± 7.49 h, ranging from 14 to 48 h. Postoperative ultrasound for all cases showed no evidence of minor or major bile leaks or CBD injuries.

**Conclusion:**

This is the first report to evaluate the use of HS as a sole instrument during LC in the pediatric age group. HS is a safe and efficient instrument that can be used alone in gallbladder dissection as well as in controlling cystic duct and artery during LC in children.

## Introduction

In spite of being one of the most common surgical procedures performed in adults, laparoscopic cholecystectomy (LC) is relatively uncommon in the pediatric age group (1). Over the past two decades, the number of LC operations in children significantly increased because gallstone disease has been increasingly recognized in children and the spectrum of pediatric biliary tract disease changed considerably. Until recently, most gallstones in children were pigmented stones caused by hemolytic diseases such as thalassemia and hereditary spherocytosis (2). Nowadays, the occurrence of gallstone disease in children has risen, principally related to the epidemic of pediatric obesity. According to a study by Pogorelic et al., the average BMI of the population under observation was substantially correlated with the number of pediatric cholecystectomies. This likely shows a link between rising obesity rates and the incidence of symptomatic cholelithiasis in children (3).

Most surgeons prefer to dissect the cystic duct using a monopolar electrosurgical hook and occlude it with simple metal clips. Alternatively, although uncommon, cystic duct ligation can be accomplished using a linear stapler, endoloops, or suture ligation (4). While the safety of using the ultrasonically-activated shears, e.g., the Harmonic scalpel (HS, Johnson & Johnson Co., Cincinnati, OH, United States), for dissection of the gallbladder is confirmed in many studies, its efficacy in the closure of the cystic artery and duct in adults is still debatable (5). The aim of our study was to study the safety and efficiency of using HS in gallbladder dissection and cystic duct control in children during LC.

## Materials and methods

This prospective study was conducted from May 2017 to April 2020 after approval by the Ethical Committee of the Alexandria Faculty of Medicine (IRB no.: 00007555, 16 February 2017). Informed consent was attained from all parents and legal guardians of the children included in the study. Children suffering from symptomatic gallstone disease were included in the study, while those having acute cholecystitis, common bile duct stones, previous upper abdominal operation, and gall bladder tumors based on radiological findings were excluded from the study.

The LC was performed in patients all under general anesthesia with the patient lying supine in the reverse Trendelenburg position with the right side up permitting gravity to assist in retraction and allowing the small bowel, to fall away from the field. Exactly four ports (three 5 mm and one 10 mm) were placed on the upper abdomen. A 5-mm port was inserted first through the umbilicus for insertion of the 5-mm 30° angle view scope. Pneumoperitoneum was established to a pressure of 10–12 mmHg. Next, a 10-mm port was inserted below the xiphoid process, where we could insert the 5 mm harmonic shear or the hook pencil via the port reducer, as well as extract the gall bladder at the end. Other two 5-mm working ports were inserted in the right flank.

**FIGURE 1:**
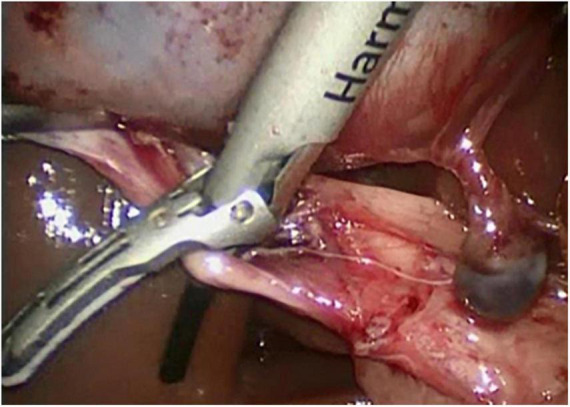
Delivery of the harmonic shear around the cystic duct.

**FIGURE 2:**
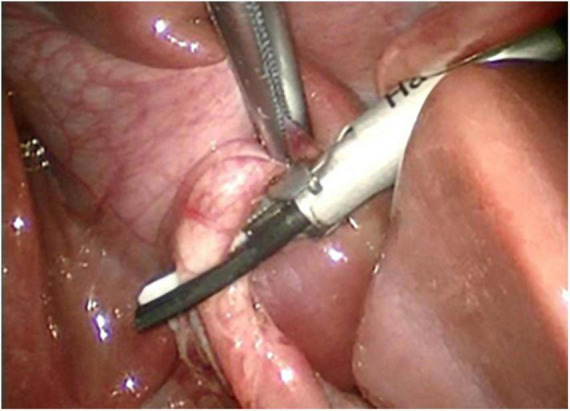
Cutting of cystic duct with harmonic shear.

**FIGURE 3:**
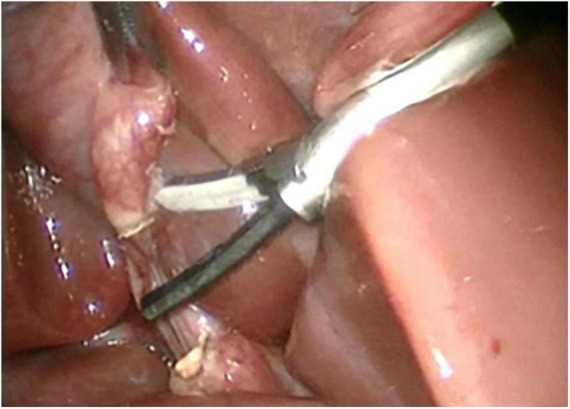
Both ends of the cystic duct after being cut by harmonic shear.

The initial step is to retract the gallbladder in order to open the Calot cystohepatic triangle and locate and skeletonize the cystic duct using Harmonic ACE^®^ + Shears (Ethicon Endo-Surgery, Inc., Cincinnati, OH, United States) at “5” power level (more cutting and less coagulation). The instrument was adjusted to power level “2” for the closure and division of the cystic duct (less cutting and more coagulation) (Figures 1, 2). To avoid damaging the common bile duct (CBD), the jaws of HS were kept at a safe distance to avoid its damage and remained closed till a click was heard and the gall bladder become detached from the cystic duct (Figure 3). All the minor branches of the cystic artery along the adjacent border of the gallbladder were cauterized. Finally, the gallbladder was dissected and removed from the liver bed, and it was sealed with a toothed crocodile 5 mm grasper through a 10mm trocar beneath the xiphoid.

The operative time, as well as any intraoperative and postoperative problems, were all recorded. Patients were examined in the outpatient clinic at the end of the first postoperative week for a clinical assessment and abdominal ultrasonography to check for any probable collections. The clinical examination and abdominal ultrasonography were repeated at the end of the first and sixth postoperative months, along with blood tests, such as bilirubin, aminotransferase, gamma-glutamyl transferase, and alkaline phosphatase levels. The primary outcome of the study was to assess the safety of using HS was assessed by searching for a biliary leak or CBD stricture. Secondary outcomes in the form of operative time, time to start oral feeding, and discharge were recorded as well.

After data was fed to the computer, IBM SPSS software package version 20 (IBM Corp., Armonk, NY, United States) was used for analysis. Number and percent were used to describe qualitative data, whilst range (minimum and maximum), mean, standard deviation, and median were used to describe quantitative data.

## Results

The present study included 42 children with symptomatic gallstone disease. A total of twenty-two patients were boys (52.3%), while 20 were girls (47.7%). Their age ranged from 3 to 13 years with a mean of 8.4 ± 3.25. The main indication for LC was hemolytic anemia in all cases except two, LC was done due to the presence of gallbladder polyps. All operations were completed laparoscopically, i.e., no conversion to open surgery was needed. The mean operative time was 40 ± 10.42 min, (range: 20–58 min. There were no intraoperative complications apart from gall bladder perforation in two cases during dissection from the liver bed. These were managed by retrieval of the spilled stones, adequate irrigation of the peritoneal cavity, and adequate antibiotic therapy. The postoperative recovery was uneventful in all patients. Patients started oral feeding after 11.3 ± 3.01 h (range: 7–18 h). The mean time of patients’ discharge was 25.47 ± 7.49 h, (range: 14–48 h). Postoperative ultrasound examination was done for all cases at the sixth postoperative month where it showed normal CBD measurements and a clear surgical bed with no minor nor major bile leaks or CBD injuries.

## Discussion

The majority of surgeons prefer to dissect the cystic duct using a monopolar electrosurgical hook and occlude it with simple metal clips in order to minimize bile leak. Nonetheless, these clips can migrate into neighboring structures, resulting in strictures due to foreign body response, act as a nidus for stone formation, and occasionally fall off leading to substantial morbidity (6, 7). Although non-popular, many surgeons prefer to ligate the cystic duct using absorbable sutures to avoid such complications; however, this adds to the length of the procedure adding a technically demanding step in order to perform the three intracorporeal sutures (8).

Ultrasonic coagulating shears were developed to allow hemostasis during laparoscopic surgery owing to their sealing effect, which is produced by coagulation of protein through high-frequency ultrasonic vibrations generating heat (9). In LC, the HS was investigated by many authors as an energy tool during dissection and removal of the gallbladder from the liver bed. What was debatable is the use of HS as a sole instrument in controlling cystic duct during LC. Bessa et al. (10) reported that the HS was as safe and effective as the more commonly used clip and cautery technique in achieving safe sealing and control of the cystic duct in the LC. Furthermore, they reported it was even superior to the latter in terms of shorter operative time and lower incidence of gallbladder perforation with subsequent bile leakage during dissection of gall bladder from the liver bed. Similar results were obtained by Westervalt (11) who reported no bile leaks in his 100 patients when the cystic duct was controlled and achieved solely by HS. In the study by Huscher et al. (12), however, bile leaks were found in 7 of the 331 patients (2.1%). All these studies were conducted on adults; nonetheless, no previous reports discussed these topics in the pediatric age group.

From our perspective, HS offers many advantages. Firstly, it serves as a 4-in-1 instrument (i.e., dissector, electrosurgical hook, a clip applier, and scissor) (13). This definitely saves time as there is no need to change instruments repeatedly. Additionally, no smoke is produced with HS; thus, no need to clean the camera repeatedly which enhances the vision during the procedure. Secondly, HS has a small area of collateral thermal injury compared to momopolar (electrocautery) or bipolar (Ligasure) diathermy as it transduces a lower amount of energy, which allows the surgeon to use the harmonic dissector adjacent to the common bile duct with no fear of CBD thermal injury or bile leakage (14, 15). Definitely, this minimizes the risk of gallbladder perforation and consequently saves time wasted in abdominal lavage and in retrieving spilled stones and reduces morbidity (16). Lastly, recent studies confirmed that, in the setting of financial restrictions encountered in low-resource countries, HS can be re-used safely without any consequences to the patient’s condition or postoperative course (17).

As regards the debate of using HS in controlling cystic ducts solely, and as claimed, by the manufacturer, the coagulation function of HS is safe when applied to vessels of up to 7 mm (18). That is why it is applied by many authors for coagulating the cystic artery as it is usually smaller than that caliber, hence, postoperative bleeding is an unexpected complication (19). In addition, after establishing the use of HS for sealing the cystic artery, some surgeons also investigated its role in sealing the cystic duct and concluded that the use of HS can be used only if the cystic duct diameter is less than 6 mm in diameter. This could be an issue in adults as cystic duct diameter can increase to more than twice the reference range in presence of cholelithiasis (20); nevertheless, in children, cystic ducts are seldom larger than 6 mm. It is worth mentioning that LC is not the first operation where the efficacy of HS was evaluated it was previously studied in other clipless laparoscopic procedures such as appendectomy or splenectomy and showed a high degree of efficacy and safety (21, 22).

In conclusion, this is the first report that studies the use of HS as a sole instrument to complete LC in the pediatric age group. HS is a safe and efficient instrument that can be used in gallbladder dissection as well as in controlling cystic duct and artery during LC in children.

## Data availability statement

The original contributions presented in this study are included in the article/Supplementary material, further inquiries can be directed to the corresponding author.

## Ethics statement

The studies involving human participants were reviewed and approved by the Ethics Committee of the Faculty of Medicine of the University of Alexandria (Alexandria, Egypt). Written informed consent to participate in this study was provided by the participants or their legal guardian/next of kin.

## Author contributions

AA and MK: data collection and manuscript writing. MA: critical revision. AK: protocol development and critical revision. All authors contributed to the article and approved the submitted version.

## Supplementary material

Supplementary Video 1 Controlling of cystic duct using HS.
